# Key Findings and Implications of a Recent Systematic Review of the Potential Adverse Effects of Caffeine Consumption in Healthy Adults, Pregnant Women, Adolescents, and Children

**DOI:** 10.3390/nu10101536

**Published:** 2018-10-18

**Authors:** Candace Doepker, Kara Franke, Esther Myers, Jeffrey J. Goldberger, Harris R. Lieberman, Charles O’Brien, Jennifer Peck, Milton Tenenbein, Connie Weaver, Daniele Wikoff

**Affiliations:** 1 ToxStrategies, Cincinnati, OH 41075, USA; 2 ToxStrategies, Asheville, NC 28804, USA; kfranke@toxstrategies.com (K.F.); dwikoff@toxstrategies.com (D.W.); 3 EF Myers Consulting, Trenton, IL 62293, USA; efmyers@efmyersconsulting.com; 4 Cardiovascular Division, University of Miami Miller School of Medicine, 1120 NW 14th Street, Suite 1124, Miami, FL 33136, USA; j-goldberger@miami.edu; 5 Military Nutrition Division, US Army Research Institute of Environmental Medicine, Kansas Street, Natick, MA 01760-5007, USA; harris.r.lieberman.civ@mail.mil; 6 Department of Psychiatry, University of Pennsylvania, 3535 Market Street, 4th Floor, Philadelphia, PA 19104, USA; obrien@mail.med.upenn.edu; 7 College of Public Health, University of Oklahoma Health Sciences Center, 801 NE 13th Street, Oklahoma City, OK 73104, USA; Jennifer-Peck@ouhsc.edu; 8 Department of Pediatrics and Child Health, Department of Community Health Sciences, Children’s Hospital, University of Manitoba, 840 Sherbrook Street, Room CE 208, Winnipeg, MB R3A 1S1, Canada; mtenenbein@exchange.hsc.mb.ca; 9 Department of Nutrition Science, Purdue University, 700 W. State Street, West Lafayette, IN 47907, USA; weavercm@purdue.edu

**Keywords:** caffeine, coffee, systematic review, pregnancy, safety

## Abstract

In 2016–2017, we conducted and published a systematic review on caffeine safety that set out to determine whether conclusions that were presented in the heavily cited Health Canada assessment, remain supported by more recent data. To that end, we reviewed data from 380 studies published between June 2001 and June 2015, which were identified from an initial batch of over 5000 articles through a stringent search and evaluation process. In the current paper, we use plain language to summarize our process and findings, with the intent of sharing additional context for broader reach to the general public. We addressed whether caffeine doses previously determined not to be associated with adverse effects by Health Canada (400 mg/day for healthy adults, 300 mg/day for pregnant women, 2.5 mg/kg body weight/day for adolescents and children, and 10 g/day for acute effects) remain appropriate for five outcome areas (acute toxicity, cardiovascular toxicity, bone & calcium effects, behavior, and development and reproduction) in healthy adults, pregnant women, adolescents, and children. We used a weight-of-evidence approach to draw conclusions for each of the five outcomes, as well as more specific endpoints within those outcomes, which considered study quality, consistency, level of adversity, and magnitude of response. In general, updated evidence confirms the levels of intake that were put forth by Health Canada in 2003 as not being associated with any adverse health effects, and our results support a shift in caffeine research from healthy to sensitive populations.

## 1. Introduction

Consumption of caffeine remains a topic of popular interest, but it is also often a cause of confusion for medical professionals, nutritionists, and the public. The editors of this special issue of *Nutrients*, related to the impact of coffee and caffeine on human health, invited us to provide a summary of the recently published article, “Systematic Review of the Potential Adverse Effects of Caffeine Consumption in Healthy Adults, Pregnant Women, Adolescents and Children”, for a broad audience. The large (64-page) systematic review was published in Food and Chemical Toxicology in April 2017, received much attention in the press, and was chosen “Best Paper of the Year” by the Editors of the journal [1]. The format of the paper followed a systematic review (SR) approach, which used an established and recognized framework that was specifically chosen to ensure transparency. Staying true to this framework required a large amount of documentation, which rendered the paper groundbreaking in terms of content but perhaps challenging to read and digest. At the same time, tracking statistics have demonstrated that the general public, in fact, has an interest in the SR findings with regard to caffeine. Scientific findings lose their value if they cannot be easily comprehended by diverse audiences. The Institute of Medicine (IOM) also recognizes this fact, and their guidance related to systematic reviews suggests that plain-language summaries can improve the work’s usability for general audiences [2]. Thus, the aim of this paper is to provide a plain-language summary of this important review, and the reader is referred to the original work for full references [1]. We hope that this approach will allow the findings to be more understandable and help individuals make educated decisions regarding their (or their patients’) consumption of caffeine. 

Caffeine (1,3,7-trimethylxanthine) is a pharmacologically active component of many foods, beverages, dietary supplements, and drugs. Interestingly, it is also used to treat very ill, often premature, newborns afflicted with apnea (temporary cessation of breathing) [3]. Caffeine is probably best recognized for its use as a flavor in cola-type beverages, and for its natural occurrence in some seeds, such as coffee and cocoa. Coffee is one of the major contributors of caffeine to the diet [4] and it has been consumed safely for centuries, as have black and green tea. Energy drinks entered the market in the 1980s, introducing another popular source of caffeine. A number of other caffeine-added products have also attempted entry into the marketplace, such as maple syrup, beef jerky, donuts, and chewing gum. These products, with varying degrees of success, have attempted to provide novel sources of caffeine to the consumer. 

The long history of caffeine use and the wide array of new products offered as sources suggest that consumers continue to desire caffeine’s pharmacological effects. In the last decades, caffeine has received both favorable and unfavorable attention from various stakeholders, such as the scientific community, the press, and Non-Government Organizations. Any general internet search yields many consumer questions related to the health and safety of caffeine. Mixed messaging in the press related to benefits and potential adverse effects, combined with the possible difficulty of assessing one’s own exposure to caffeine, can lead to a great deal of uncertainty for the consumer. To address this concern in the United States, health-care professionals made a public request in the form of a letter to the FDA to gather data related to overall caffeine safety [5]. As part of this request for more investigation, the IOM’s Food and Nutrition Board and Board on Health Science Policy hosted a two-day workshop in August of 2013, entitled, “Caffeine in Food and Dietary Supplements: Examining Safety”. This workshop provided a public forum for discussion and examination of the potential health hazards of caffeine, which were later summarized in a large (190-page) publication [6]. The bulk of the data presented at that time came from the Oak Ridge National Laboratory (ORNL) report that was commissioned by the FDA [7]. The IOM’s public forum event to discuss caffeine safety was not unprecedented—in the past couple of decades, many other countries have initiated discussions about the use of caffeine in food and beverages, with the intent of better understanding the consumption practices and potential safety concerns (India [8]; Australia and New Zealand [9], Europe [10], and Canada [11]). The European Food Safety Authority (EFSA) has the most recent publication of such an effort [10]. 

Most of the authoritative reviews or discussions mentioned above allowed for some sort of public and stakeholder input, either via submission of public comments directly or participation in public forums for discussion, and three major themes or requests continually surfaced: (1) help the consumer understand how much caffeine is actually in food and beverages (exposure); (2) help the consumer understand what level of caffeine is safe (risk); and (3) better elucidate what sort of adverse effects are associated with particular doses (dose-effect). Throughout the discussions and various publications, another commonality was the repeated references to one particular publication—Nawrot et al. (2003) [11]—and subsequent references to the suggested “safe values” for ingestion of caffeine those authors put forward. 

Nawrot et al. (2003) [11] is a peer-reviewed publication from Health Canada, which conducted a narrative, but not systematic, review of scientific literature. We believe that at least part of the reason this article has been so heavily cited is that it is easy to read and covers multiple areas of interest related to caffeine. In developing their conclusions, Nawrot et al. (2003) [11] reviewed many potential adverse-event areas; however, given the voluminous scope, they focused primarily on five outcomes (1) acute toxicity (defined herein as abuse, overdose, and potential death); (2) cardiovascular; (3) bone and calcium; (4) behavior; and (5) development and reproductive toxicity. The authors also touched on genotoxicity, mutagenicity, and carcinogenicity, but these have not been a focal point of concern for caffeine outside of reproductive toxicity. The authors concluded after conducting their qualitative review that the consumption of up to 300 mg/day for pregnant women and 2.5 mg/kg body weight/day for children is not associated with adverse effects. They went on to conclude that an intake dose of up to 400 mg caffeine/day is not associated with adverse effects in healthy adults [11]. Importantly, since Nawrot et al. [11] was published in 2003, more than 10,000 papers on caffeine-related topics have been published, and of those, more than 5000 address effects or exposure in humans. In addition, 800+ reviews related to various human health effects of caffeine have also been published (nearly all are specific to a particular adverse endpoint category). 

With this as background and in light of the wealth of new data in the peer-reviewed literature, and because Health Canada’s work is so commonly referenced in discussions and debates over caffeine safety, the goal of our systematic review was to investigate whether or not the Nawrot et al. (2003) [11] conclusions remain current as an acceptable level of protection to the healthy general public. We chose the same outcomes for evaluation, because these endpoints reflect importance, as documented in other comprehensive evaluations [6,10,11,12,13], and indicate stakeholder interest. Therefore, it is useful to determine whether the values that were put forth by Nawrot et al. (2003) [11] remain appropriate and as such can still serve as a basis to assure the typical healthy caffeine consumer of a reasonable certainty of no harm. This evaluation also allows scientists to move on from this question and focus more on sensitive subpopulations that may be at greater risk. 

Thus, the need for our systematic review was established. Specifically, our objective was to determine whether the literature published since the 2003 Health Canada review supports the conclusion that caffeine consumption at amounts up to 400 mg/day for healthy adults, 300 mg/day for healthy pregnant women, and 2.5 mg/kg body weight/day for healthy children is not associated with adverse effects. We also evaluated the consumption of 2.5 mg/kg body weight/day in adolescents, although this was not specifically addressed by Nawrot et al. (2003) [11]. 

## 2. Materials and Methods 

**Figure 1:**
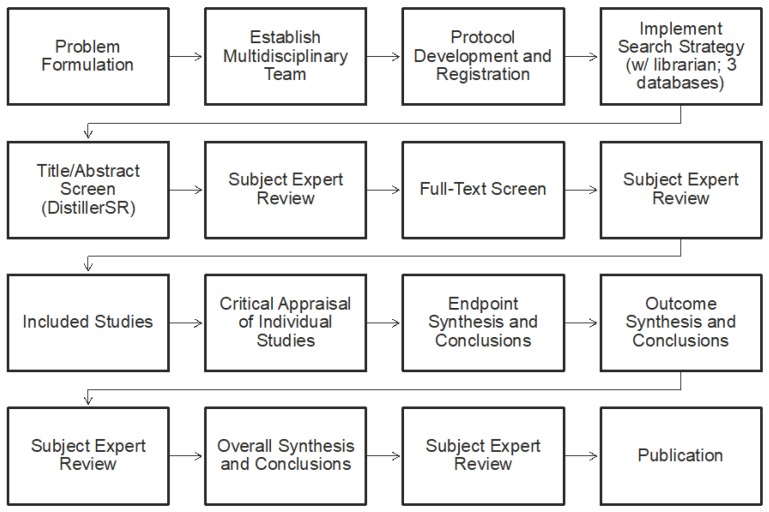
Work flow of the systematic review.

The Systematic Review (SR) was conducted using the IOM’s *Finding What Works in Health Care—Standards for Systematic Reviews* as guidance [14]. The overall work flow of the systematic review is shown in Figure 1 and it included problem formulation; developing a protocol; conducting a systematic search (informed by a librarian) of three databases; screening of literature for inclusion/exclusion; critically appraising individual studies; conducing endpoint, outcome, and overall syntheses and weight-of-evidence analyses; and, reporting the systematic review. 

Consistent with IOM recommendations, the first step that is involved establishing a team with appropriate expertise and experience (Table 1). The project team was composed of eight scientists from ToxStrategies with a range of expertise, as well as a scientific advisory board (SAB), of which each member had expertise in an outcome (e.g., cardiovascular) evaluated in the review. 

**Develop the Population Exposure Comparator Outcome (PECO).** As part of the IOM framework problem formulation, the specific research question or objective addressed in the systematic review was based on a “PECO” format (which is different from the PICO (population, intervention, comparator, and outcome) format that is often used in nutrition and clinical medicine). Specifically, the PECO was:

“For (population), is caffeine intake above (dose), compared to intakes (dose) or less, associated with adverse effects on (outcome)?” As an example, for healthy adults, the PECO would be, “For healthy adults, is caffeine intake above 400 mg/day, compared to 400 mg/day or less, associated with adverse cardiovascular effects?”

**Figure 2:**
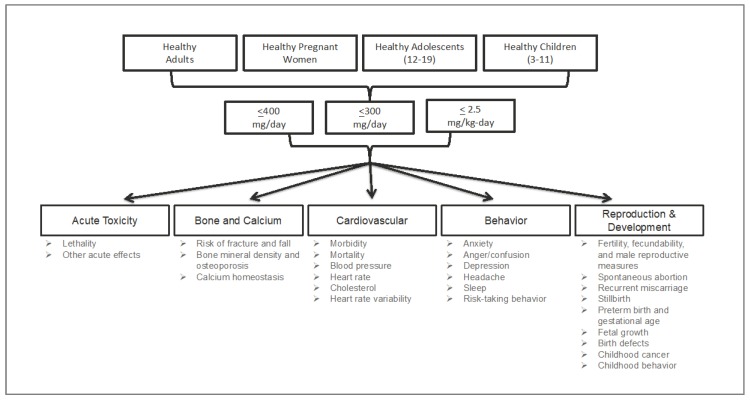
Populations, dose/intake levels, outcomes, and endpoints evaluated.

The SR focused on five outcomes (Figure 2): acute, cardiovascular, bone and calcium, behavior, and development and reproduction (further descriptions of the endpoints included within each of these outcomes can be found in the results section of each outcome. It should be noted and emphasized that, within each outcome (e.g., cardiovascular), there were many endpoints (e.g., morbidity, mortality, blood pressure, heart rate, etc.)*.* A sixth outcome, pharmacokinetics (PK), was included as a contextual topic; the objective was to generally characterize the current understanding of caffeine kinetics and critically review any information that advances the science. Thus, this topic particularly pertained to the differences and similarities between our populations of interest, characterization of kinetics in children and adolescent populations of interest, and characterization of kinetic parameters (particularly fast/slow phenotypes) in the context of the outcomes of interest. 

Four populations were evaluated: healthy adults, healthy pregnant women, healthy adolescents (aged ≥12–≥19 years), and healthy children (aged ≥3–<12 years). For all outcomes, except acute, the daily intake (exposure) values that were evaluated were based on those established by Nawrot et al. (2003) as acceptable levels of daily intake. Thus, the exposure values (the “E” in the PECO) were 400 mg/day (10 g for acute), 300 mg/day, and 2.5 mg/kg body weight/day for adults, pregnant women, and adolescents and children, respectively. Similarly, comparators (the “C” in the PECO) were ≤400 mg/day for adults (10 g for acute), ≤300 mg/day for pregnant women, and ≤2.5 mg/kg body weight/day for adolescents and children. Thus, for example, we investigated whether the literature supports a finding that a daily exposure of 400 mg caffeine per day is safe for adults (the exposure), or rather, whether the literature supports the safety of daily exposures to less than 400 mg caffeine body weight per day for adults (the comparator). 

**Protocol Registration.** Consistent with expectations for transparency as part of the framework, a protocol for each outcome was developed and registered on PROSPERO (PROSPERO protocol nos. CRD42015026704, CRD42015027413, CRD42015026673, CRD42015026609, and CRD42015026736; https://www.crd.york.ac.uk/PROSPERO/). Each protocol included: (1) context and rationale for the review; (2) study selection and screening criteria; (3) descriptions of outcome measures, time points, and comparison groups; (4) search strategy; (5) procedures for study selection; (6) data extraction strategy; (7) approach for critically appraising individual studies; and (8) method for evaluating the body of evidence. The objective of registering a protocol is to make the approach apparent a priori, as is consistent with the IOM guidelines and standard practice of systematic review. 

**Literature Search.** A comprehensive search strategy was iteratively developed and employed with the assistance of a librarian who had expertise in the conduct of SRs. Three databases were searched: PubMed, EMBASE, and the Cochrane Database of Systematic Reviews. DistillerSR (a software tool that facilitates systematic review) was used for screening and selecting studies, as well as for documenting the extraction and evaluation of data. It is important to note that, to be included in the SR, studies had to provide a quantitative estimate or measurement of individual exposure to a caffeine source associated with an adverse effect. We included many forms of caffeine, such as coffee, tea, chocolate, cola-type beverages, energy drinks, supplements, medicines, and energy shots. For included studies, basic information that was reported by the author was extracted from each study (i.e., direct extraction of information from the text), along with other selected information needed to inform the PECO questions (e.g., dose/exposure calculations) that may have required interpretation by the analysts. For example, the exposure (dose) of caffeine was extracted directly from the studies when the authors of the studies evaluated caffeine directly or reported findings based on the amount of caffeine in given sources. In cases where this was not directly reported, the reviewers standardized the quantity of caffeine; this process was explained in supplementary materials to the original publication, and the interested reader can find more details there. 

**Individual Study Evaluation.** During extraction of information from an individual study, the level of adversity (potential for harm) of the endpoints within the study was characterized [15]. That is, the reviewer noted whether the study evaluated a clinical (e.g., morbidity or mortality) or physiological endpoint (e.g., blood pressure changes), as well as the importance of the effect for decision making (e.g., mortality vs. blood pressure changes). Additionally, from each study and each eligible endpoint within a study, specific values were selected or determined in order to compare to the PECO (i.e., the conclusions of Nawrot et al., 2003 [11]). This involved identifying effect and no-effect levels. Specifically, we endeavored to establish a lowest-observed-effect level (LOEL), or, preferably, a no-observed-effect level (NOEL) (e.g., a daily exposure of X caffeine/day was without effects on Y endpoint in study Z), which could then be used for comparison to the PECO. 

**Figure 3:**
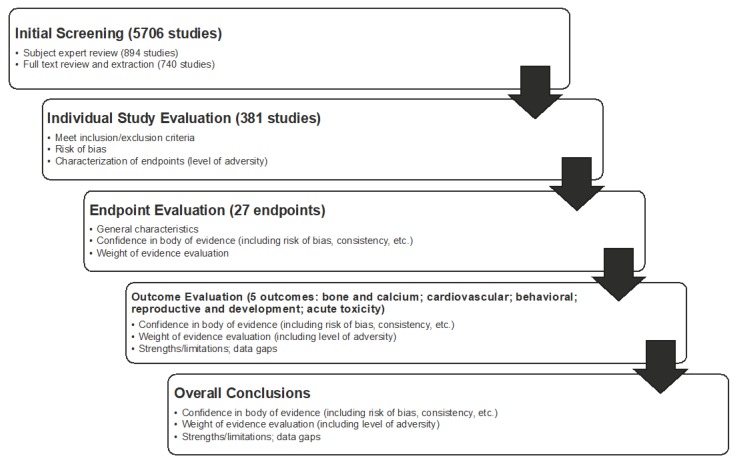
Review process, from initial evaluation to reaching overall conclusions.

Following data extraction, individual studies were assessed for the risk of bias (internal validity) using the National Toxicology Program’s Office of Health Assessment and Translation (OHAT) Risk of Bias Rating Tool for Human and Animal Studies [15]. Bias is differentiated from the broader concept of quality of the methodology and is aimed at assessing the systematic error—a measure of whether the design and conduct of a study compromised the credibility of the link between exposure and outcome [14,15,16]. This approach evaluated what are called “specific domains” based on study type (i.e., controlled trial vs. observational study). Specific domains related to bias included selection, confounding, performance, detection/measurement, attrition/missing data, reporting, and other types of bias. Each domain was rated from “definitely low risk of bias” to “definitely high risk of bias” per the OHAT tool. These ratings for individual studies were then considered in the weight-of-evidence assessment when developing conclusions for the endpoint, outcome, and overall (Figure 3). 

**Determination of Weight of Evidence.** Following the appraisal of individual studies, the body of evidence was evaluated using a weight-of-evidence approach for each endpoint, each outcome, and overall (Figure 3). Similar to the approach and conclusions of Nawrot et al. (2003) [11], the objective in the weight-of-evidence assessment was not to find the most protective amount or the lowest amount associated with an effect, *per se*, but rather, to make a determination that is based on the body of evidence as a whole, which included considerations for positive and negative findings, quality of data, level of adversity, consistency, and magnitude of effect (for studies with effects below the comparator). The weight-of-evidence approach implemented was based on the framework established by the IOM [14] and it was complemented by guidance from the National Toxicology Program handbook on systematic reviews [17], given the specific application to toxicological assessments. We also relied on the GRADE (Grades of Recommendation, Assessment, Development and Evaluation) process in determining and implementing our weight-of-evidence approach [18,19]. 

In evaluating and conducting a qualitative synthesis of the body of evidence, data were described based on the volume of data above and below the comparator, as well as the types of effects and quality of evidence of data that are above and below the comparator. An initial level of confidence in the evidence was assigned based on key features of study design: controlled exposure, exposure prior to outcome, individual outcome data, and comparison group used [17]. Then, using expert judgement, a number of additional factors were considered for the overall body of evidence, which yielded increases or decreases in the confidence level. These factors included the following: overall risk of bias, indirectness (when the population, exposure, or outcome differ from those in which we were interested), magnitude of effect, confounding, and overall consistency [17,18,19]. Consideration of endpoint importance in terms of the endpoint’s degree of adversity [18,19] was also important in reaching weight-of-evidence conclusions. 

**Figure 4:**
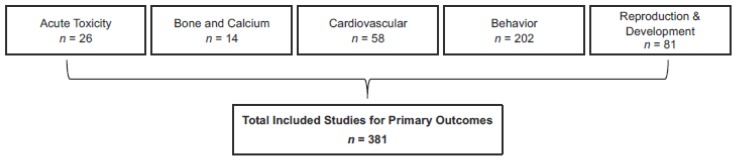
Number of studies that met the SR inclusion criteria and were reviewed for each endpoint.

**Weight-of-evidence determinations** were made by endpoint, outcomes, and overall (Figure 4). Such determinations were also made by population, because the comparators were different for healthy adults, pregnant women, and children. Conclusions were developed by categorizing evidence relative to the comparator (an intake value not associated with adverse effects) as follows: comparator is acceptable (i.e., evidence supports the Nawrot et al., 2003 [11], conclusions regarding intake), comparator is too high (i.e., evidence suggests the comparator is too high for a given endpoint), or comparator is too low (i.e., evidence suggests the comparator could be higher for a given endpoint). Using a similar approach, conclusions were also developed for the outcome. When developing outcome conclusions, clinical endpoints with a high level of adversity were given the most weight. Several tools were used to facilitate and support the weight-of-evidence evaluation, including generation of evidence tables, risk-of-bias heat maps, summary plots of selected NOEL/LOEL data from individual studies, and a tabular summary of the confidence in the evidence for each outcome and endpoint. Conclusions were not developed for endpoints that contained fewer than five studies; in these instances, summary thoughts were provided, but data were determined to be insufficient to reach a conclusion. 

Transparency in Reporting. All data from the systematic review were placed in a freely available Agency for Healthcare Research and Quality (AHRQ) Systematic Review Database Repository (SRDR). 

## 3. Results

**Figure 5:**
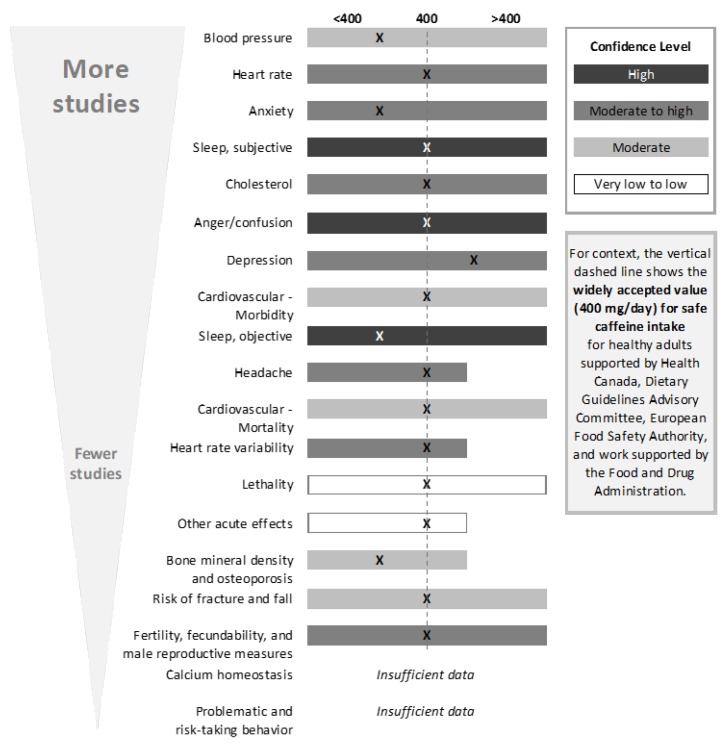
Summary of the spectrum of data and our endpoint conclusions for healthy adults, weighted for level of confidence in the body of evidence considering risk of bias, magnitude, consistency, and other factors. Shading indicates that data reported effects at the corresponding intake level (<400, 400, or >400 mg caffeine/day), and darker shading indicates increased confidence in the body of evidence (from very low to high). X indicates the SR weight-of-evidence conclusion for the level of intake not associated with significant health effects. Although effects were observed at exposures below 400 mg (e.g., blood pressure, bone mineral density and osteoporosis), these results did not affect the overall conclusion of the SR, due to considerable variability in individuals’ sensitivity to caffeine and potential confounding, and the effects were limited to physiological effects following acute exposure, and subgroups of clinical endpoints, such as those with low calcium intake. Such effects were generally of low magnitude, and/or were of overall low or negligible consequence to downstream effects. Several studies also showed a lack of effects on clinical endpoints at exposures above 400 mg.

Throughout this section, the reader is reminded to refer to the original paper for extensive references [1]. This approach (not including full references here) was chosen to best fulfill the goal of simplifying the text so that this summary can accomplish its aim—i.e., to provide ease of reading and understanding for diverse audiences. Figure 5 below summarizes the key findings from each outcome, as well as perspective that is related to confidence in the value based on our analysis. The manner in which these conclusions were reached is discussed in each section for the respective outcomes below. 

### 3.1. Literature Searching

All databases were searched on 8 June 8 2015. Following removal of duplicates, 5706 records of human studies were identified. Following committee reviews, internal quality-control efforts, and SAB review of title and abstract screening, 740 records were carried forward to full text review (Figure 3). The most common reasons for exclusion during title and abstract review were as follows: outcomes not included in the SR (e.g., cancer), unhealthy populations, co-exposures (e.g., alcohol), study was focused on benefit or therapy, and in vitro studies. Following a full text review, a total of 381 studies (plus 46 for contextual pharmacokinetic discussion) were included in this SR relevant to the five outcomes considered for healthy adults, children, adolescents, or pregnant women. Almost half of the studies (42%) specifically evaluated caffeine as a source; the majority of the remaining studies evaluated coffee (21%), tea (12%), and soda (9%) as sources of caffeine, whereas the other studies evaluated caffeine from energy drinks, chocolate, medicine, and other sources. In 77% of the studies, the exposure (dose) of caffeine did not need to be standardized (i.e., the author either evaluated caffeine directly or reported findings based on the amount of caffeine in the given sources). With respect to study type, more than half of the studies (63%) were controlled trials. The remaining were observational studies as follows: cohort studies (14%), case-control studies (9%), cross-sectional studies (5%), and meta-analyses (2%). Seven percent of the publications were case reports or case series, all of which were associated with the acute outcome (these were excluded for other outcomes). The majority of the literature (79%) identified and reviewed the involved adult populations. Literature characterizing the outcomes of interest in other populations was much more limited, including studies that involved pregnant women (14%), adolescents (aged 12–19 years) (4%), or children (aged 3–11 years) (2%). Data were extracted by the research team and rated for risk of bias and indirectness (internal and external validity). Selected no- and low-effect intakes were assessed relative to the population-specific comparator. See Figure 4 for the specific number of studies reviewed per outcome. 

### 3.2. Endpoint Evaluation

Results by outcome are discussed below. Often, observational studies relied on food frequency questionnaires and thus used categorical exposure groups based on self-reported exposure (e.g., <1 cup/day, 1–3 cups/day). Thus, the studies that directly evaluated caffeine (i.e., low level of indirectness) were given more weight in the body-of-evidence assessment relative to those that evaluated caffeine via the consumption of coffee or other substances, such as soda, tea, and chocolate, which needed to be standardized by the reviewer. It should also be noted that the general lack of mention of pregnant women in each section, outside of the outcome for reproductive effects, is a result of the lack of studies investigating this subpopulation. Figure 5 is a graphical depiction of the key findings discussed below. 

To deliver the key findings from the original work in an easy-to-follow format, we have chosen to omit the original references that are cited extensively in the SR. However, the reader will find that the summary format follows that of the original text, and full references can be found therein: Food and Chemical Toxicology 109 (2017) 585–648 [1].

#### 3.2.1. Bone and Calcium 

The potential for caffeine to adversely affect bone metabolism was raised in Nawrot et al. (2003) [11], and this was likely considered as an area of concern due to work that originated in the 1980s in the lab of Heaney and Recker [20]. This work examined the effect of caffeine on the calcium economy in the bone, and concerns regarding risk of osteoporosis followed soon after. Because this was an important outcome of interest raised by Nawrot, we specifically looked for literature that investigated the relationship between caffeine and risk of fracture and fall, bone mineral density (BMD) and osteoporosis, and metabolic impacts on calcium homeostasis. The majority of the studies reviewed evaluated associations between caffeine consumption and BMD or bone mineral content (BMC); in some studies, these data were also used to characterize osteopenia. Results were found to vary by bone site. Overall, there were 14 studies that met the inclusion criteria, because they permitted comparison to the conclusions of Nawrot et al. (2003) [11]. Most studies were observational (including large cohorts, such as the Nurses’ Health Study), although randomized controlled trials were included as well, and the study populations were healthy adults (with the exception of one study that also included adolescents). 

In reviewing studies for this outcome, we recognized that calcium intake was a potential confounding factor that was not accounted for equally in all studies. Effects of caffeine on bone are most often associated with increased urinary calcium excretion. Altered calcium balance through perturbing calcium excretion can influence bone mass. However, urinary calcium excretion is affected by calcium intake, so calcium intake needed to be considered in the analysis. This was reported by the aforementioned Heaney and Recker (1982) [19], the research group that first identified caffeine as a potential risk; however, they later concluded that individuals who ingest the recommended daily allowance of calcium are not at risk of effects from caffeine on calcium economy of the bone (Heaney, 2001) [21]. To this end, it is noted that almost 20% of the United States (US) adult population does not consume the estimated average requirement of calcium [22]. Other important common variables accounted for in studies included age, weight, body mass index (BMI), other nutrient intake, alcohol consumption, smoking habits, and physical activity level. 

Exposures evaluated in the evidence base ranged from below 20 mg/day up to 760 mg/day. For risk of fracture and fall, the majority, but not all, of the data demonstrated a lack of effects at levels below and well above (up to 760 mg/day) the comparator of 400 mg/day, with a moderate level of confidence. It is worth noting that there was no significant concern for those with adequate calcium intake. For BMD and osteoporosis, the majority of studies reviewed support a finding that the comparator of 400 mg/day in healthy adults is not harmful, although more evidence is needed for effects of caffeine intake above the comparator, because only one study examined such exposure. Calcium homeostasis was also reviewed, but only two studies met the inclusion criteria, and thus, no conclusion was developed. No data for children, adolescents, or pregnant women were available. 

Weight of Evidence for Outcome. Overall, the recent evidence is consistent with the conclusions reached by Nawrot et al. (2003) [11] for bone and calcium endpoints. Individual studies generally had a low risk of bias. When the weight of evidence was considered, 400 mg/day was found to be an acceptable intake that should not cause concern with regard to adverse effects on bone or calcium-related endpoints, particularly when individuals are consuming adequate amounts of calcium. When effects were observed at levels below 400 mg/day, they were physiological effects that followed an acute exposure, or they occurred in population subgroups; and they were generally of low health impact Limitations of the data included uncertainty in exposure estimates, ambiguity regarding calcium intake, and a high level of indirectness. Due to factors such as the consideration of only females and only one site (as opposed to fracture risk at all sites evaluated), as well as the use of different consumption groupings by study authors, the uncertainty associated with assessing caffeine exposure (particularly relative to calcium consumption), and the lack of consistently observed effects (above or below the comparator), a moderate to low level of confidence was placed on this conclusion. 

#### 3.2.2. Cardiovascular

Caffeine is a central nervous system stimulant, and its pharmacological activity involves non-specific antagonism of the adenosine receptor, which in terms of the cardiovascular system, produces various effects [23]. For that reason, extensive literature reports both caffeine’s acute effects (e.g., blood pressure, heart rate) and its chronic effects (e.g., heart disease) on this system. With this background, we considered the effects of caffeine on mortality, morbidity, blood pressure, heart rate, cholesterol, and heart-rate variability. A key factor in evaluation of endpoints other than mortality and morbidity was the consideration of level of adversity, or how much a measured endpoint actually affects a person’s overall state of health, in both the short and long term. For example, elevated heart rate, while considered an “adverse effect”, is a temporary state, and occasional increases in heart rate do not affect one’s overall health status. 

Overall, there were 203 studies that, after full review, met the inclusion criteria of the SR, because they permitted comparison to the conclusions of Nawrot et al. (2003) [11]. A large majority of the included studies were randomized controlled trials (RCTs), and the remaining were observational studies, meta-analyses of observational studies, and one meta-analysis of RCTs. Exposure was well defined in the RCTs, with most studies administering pure caffeine in pill/capsule or liquid form in a single “acute” exposure or dose, which meant a high level of directness. Often in the clinical studies, participants had fasted or abstained from caffeine consumption for some number of hours or an entire day before exposure. Some study designs involved pre-treating individuals, followed by a challenge of caffeine. Most studies involved healthy adult populations, while only 11 involved children or adolescents; however, not enough evidence existed for children to reach an overall conclusion for that population. Most of the controlled trials evaluated few, if any, potential confounders, whereas the majority of the observational studies included analyses accounting for many common risk factors for cardiovascular disease (CVD) (e.g., age, sex, smoking, alcohol consumption, BMI). 

Quantified exposures generally ranged from below 50 mg/day to more than 800 mg/day. About one-half of the data points were below the comparator of ≤2.5 mg/kg body weight in studies of children and/or adolescents. There was a moderate to high level of confidence, depending on the endpoint. The endpoint of cardiac mortality was reviewed, and the majority of evidence supports a conclusion that 400 mg caffeine/day in healthy adult populations is an acceptable intake that is not associated with significant concern. Even at higher intakes, up to ~822 mg/day, there are no consistently reported effects on mortality; further, several studies reported findings that suggest protective effects. Regarding cardiovascular morbidity, when all data were considered collectively, and considering the greater utility of meta-analyses, evidence supports that 400 mg caffeine/day in healthy adult populations is an acceptable intake that is not associated with significant effects for this endpoint. Some studies, including two meta-analyses, reported a lack of effects above the comparator (suggesting that the comparator is too low). In several cases, associations were observed only in specific genotypes, highlighting the potential role of kinetic influence on pharmacodynamics (PD; discussed below in the pharmacokinetics section). No data were available for pregnant women, adolescents, or children. 

Blood pressure was a heavily studied endpoint, with more than 100 controlled trials using exposures ranging from 50 mg to 1 g/day and considering different aspects of blood pressure. It is important to note that chronically elevated blood pressure is a known risk factor for CVD [24], whereas intermittent blood pressure elevations, such as those that are associated with exercise, are not. Taken together, studies were relatively consistent in demonstrating that exposures to caffeine at intakes both below and above the comparator of 400 mg/day have the potential to minimally increase blood pressure (often only a few mmHg) in all the populations evaluated. The biological significance of this small magnitude of change is difficult to interpret relative to the determination of adversity, because such a determination is likely to be conditional. When the evidence is considered collectively, findings suggest that the comparator of 400 mg/day in healthy adults is too high if one is considering only the potential for caffeine to cause a physiological change in blood pressure (which may or may not be adverse). However, when considering the small magnitude of changes in this physiological parameter, as well as the lack of information demonstrating an association between chronic caffeine-mediated blood pressure increases relative to known cardiovascular risk factors, the comparator of 400 mg/day is likely acceptable with a moderate to high level of confidence. Regarding the comparator of 2.5 mg/kg body weight/day in children, findings were mixed with regard to changes in blood pressure (but as noted above, blood pressure changes may not necessarily be adverse). As in the healthy adult population, when considering the small magnitude of changes and the lack of association between chronic caffeine-mediated blood pressure increases and known cardiovascular risk factors, evidence shifts to support the comparator of 2.5 mg/kg body weight/day with a moderate to high level of confidence. Additionally, results indicate that it would be prudent to evaluate blood pressure in children and/or adolescents with significant caffeine intake and consider limiting such intake for those with significant caffeine-mediated blood pressure rise. There were no data for pregnant women. 

Mainly controlled trials evaluated heart rate, with exposures ranging from <100 to 780 mg of caffeine/day, often evaluated during exercise. Collectively and with a moderate to high level of confidence, data supported that the comparator of 400 mg caffeine/day in healthy adults is acceptable in terms of not raising meaningful concern regarding the adverse effects on heart rate. Heart rate was often, but not always, significantly increased during or after exercise at a wide range of caffeine exposures, with the reported increase in these studies considered to be a beneficial (i.e., performance-enhancing) effect (heart-rate increase during exercise is a key mechanism to improve cardiac output). For children and adolescents, the data support a relationship between caffeine exposure and decreased heart rate; however, further characterization of exposures associated with such an effect were difficult, given that changes were observed in studies both below and above the Nawrot et al. (2003) [11] comparator of 2.5 mg/kg. Thus, it was determined that the evidence base was insufficient to render a conclusion regarding appropriateness of the comparator for potential impacts of caffeine consumption on heart rate in children and adolescents. There were no data for pregnant women. 

Caffeine effects on cholesterol were investigated in controlled trials, with exposures ranging from 180 to 475 mg caffeine/day; relatively consistent data showed a lack of effect of caffeine consumption on cholesterol at intakes below and above the comparator. This supports a conclusion that, for cholesterol, 400 mg/kg is an acceptable comparator in healthy adults, with a moderate to high level of confidence. No data were available for pregnant women, children, or adolescents. 

Heart-rate variability (HRV) was the final endpoint evaluated in the category of cardiovascular effects, with a moderate to high level of confidence. Exposures ranging from 40 to 500 mg caffeine/day were investigated in controlled trials, and most subjects were habitual consumers of caffeine or coffee, whereas others were relatively caffeine naïve or not specified. Taken together, there was no consistent effect of caffeine on HRV at intakes below or above the comparator, thus supporting that 400 mg caffeine/day in healthy adults is an acceptable intake that is not associated with significant change in heart-rate variability. 

Weight of Evidence for Outcome. Overall, the recent evidence is consistent with the conclusions of Nawrot et al. (2003) [11], and we maintain a moderate to high level of confidence in the evidence base. Most of the studies were clinical trials that were designed to specifically evaluate caffeine, so the level of indirectness was low. When the weight of evidence was considered, 400 mg/day was concluded to be an acceptable intake that is not associated with significant concern for adverse cardiovascular health effects in healthy adults. In general, evidence for clinical endpoints (mortality, morbidity) indicated that 400 mg/day is too conservative, and consuming higher amounts of caffeine would still be safe. While effects were seen for physiological endpoints (e.g., blood pressure, heart rate) at intakes below 400 mg/day, it remains unclear what amount of change would be considered adverse in a clinical or toxicological context. Data in children and adolescents were limited to 11 studies that evaluated physiological endpoints. Therefore, it was determined that the evidence base was insufficient to render a conclusion regarding the appropriateness of the comparator for assessing the potential impacts of caffeine consumption on cardiovascular outcomes in these populations. The available data for blood pressure and heart rate are inconsistent; several studies that report physiological changes are described below. 

#### 3.2.3. Behavioral

As discussed in the Pharmacokinetics/Pharmacodynamics section of this article, caffeine is probably best known for two of the behavioral effects it exerts on the body through antagonism of the adenosine receptor: increasing mental alertness and vigor. Although it may seem remiss to not include these effects here, because this systematic review was intended to look only at potential adverse effects, these mood states were not relevant to the inclusion criteria. Instead, the main categories that encompass potential caffeine-related adverse effects were mood, withdrawal, headache, and sleep, which were similar to those that were described in Nawrot et al. (2003) [11]. One newer category that was not covered in Nawrot et al. (2003) [11] was that of “risk-taking behavior”, which has become a topic of heightened interest in adolescents and young adults with the rise in popularity of energy-drink consumption in these cohorts. 

After full review, 80 studies met the inclusion criteria of the SR, because they permitted a comparison to Nawrot et al. (2003) [11] conclusions. The majority of these were RCTs with healthy adults. For sensitive populations, only five studies were found that met the requirement for quantitative information; these studies were conducted in children or adolescents, and no studies in pregnant women met the criteria. In the controlled trials, a large number administered pure caffeine, which led to a low level of indirectness. 

As has been described elsewhere in this summary, confounding remains an important consideration. For the endpoint of behavior, confounders, such as smoking, age, and sex, and sometimes anxiety sensitivity or sleep behavior, were taken into consideration by the authors, depending on the endpoint objective. Most studies evaluated caffeine intake that fell at or below the comparator of 400 mg/day, with a quantified exposure range from 60 mg/day up to approximately 1.2 g/day. Overall confidence in this data set was moderate to high. 

A number of endpoints represented potential behavioral effects, and for this reason, major categories were used for simpler designations, and subdivisions within each category were discussed. For example, the category of “mood” was subdivided to include anxiety and other general mood states. In studying this endpoint, the majority of studies were randomized controlled trials, and within the study design, questionnaires, such as the Profile of Mood States (POMS) or visual analogue scales (VAS), were frequently used to summarize perceptions by subjects. Using this form, subjects could use common terms such as vigor, depression, fatigue, anger, and confusion, as well as anxiety, to gauge their mood state. It is important to note that these dimensions represent nonclinical mood states, and changes to them do not necessarily indicate negative effects. In our review, we also wanted to note (as did Nawrot et al., 2003 [11]) that, when evaluating anxiety, some of the potential associated manifestations, such as “tension”, “jitteriness”, “nervousness”, and “worry”, must be also considered in light of caffeine’s pharmacologic ability to increase alertness and arousal, and thus, these can be associated effects. Taken together, some but not all evidence, primarily from RCTs involving single/short-term caffeine exposure (range 70–1200 mg caffeine/day) and subjective measures of anxiety, suggests that the comparator of 400 mg/day can lead to increases, albeit small, in measures of anxiety in adults. There were no data for pregnant women.

Tolerance to the stimulant effects of caffeine occurs with repeated dosing over several days, and this explains why the effects on increased blood pressure are largely temporary and not usually clinically important in the long term. The opposite of tolerance is withdrawal, which is reported as sleepiness and fatigue if the usual dose of caffeine is omitted for a day. This has been clinically recognized by the diagnosis of “caffeine withdrawal” by the American Psychiatric Association (DSM-5, p. 506) [25]. 

“Anger” and “confusion” were other subdivisions of mood for which a number of RCTs used doses ranging from 70 to 1200 mg caffeine. Confusion included difficulty concentrating and bewilderment or muddled perception. Overall, the data suggest that the comparator of 400 mg/day is an acceptable daily intake that is not associated with significant concern regarding anger and confusion. There were mixed findings when doses were administered above the comparator—well-rested individuals manifested no effect, but at very high doses (1200 mg/day given as 400 mg 3×/day for seven days), there was a significant increase in POMS anger scores. There were no data for pregnant women. 

Depression and related endpoints were investigated in mostly RCTs, but also a fair number of observational studies where exposures ranged from 80 to 1200 mg caffeine/day. Similar to Nawrot et al. (2003), the finding from our review indicated no effects of caffeine, even at very high exposures, on scores of depression. Taken together, the weight of evidence suggests with moderate to high confidence that the comparator of 400 mg/day of caffeine is an acceptable intake. A few studies indicated a decreased risk of depression effect that is associated with exposure to caffeine. There were no data in pregnant women. 

Headache was another category of relevance and interest, due to both “acute” effects and potential “withdrawal” effects of caffeine. Ratings of headaches (pain or severity), which are often captured via customized questionnaires or a VAS, were not significantly increased in any of the controlled trials that evaluated the effect of acute caffeine ingestion doses below the comparator of 400 mg. 

For adults, the weight of evidence supports, with a moderate to high level of confidence, that consumption of ≤400 mg caffeine is not associated with an increase in headaches. However, like the evidence presented in Nawrot et al. (2003), observational studies do indicate a potential link between caffeine use and headache prevalence in some individuals, although some of this effect is likely due to withdrawal-related symptoms. There were no data for pregnant women. 

Sleep was a category divided by subjective and objective categories, because the types of endpoints evaluated by each metric vary (i.e., different endpoints of sleep). The subjective effects are those that looked at perceptions of “sleepiness”—mood states, such as fatigue, tiredness, drowsiness, or weariness that are often measured with POMS or VAS questionnaires. Objective measures included sleep latency, duration, and efficiency, all of which are quantitated for the night(s) following caffeine intake. Of the large number of controlled trials that were reviewed, the majority demonstrate that the comparator of 400 mg caffeine/day is acceptable as an intake that is generally not associated with concern regarding adverse effects on sleep. There were a few cases in which prolonged dosing was associated with increased fatigue, but the magnitude of these changes was difficult to assess. Caffeine’s mode of action in the central nervous system (CNS) helps, in part, to explain why most caffeine doses tested in these studies may indeed provide some benefit on this endpoint by reducing perceived fatigue; however, higher doses might disrupt sleep and lead to an increase in fatigue when consumed over the course of several days. 

Objective effects of sleep were evaluated in controlled studies and observational studies. With respect to the data obtained via objective measures of sleep in adults, results indicate that the comparator of 400 mg caffeine/day is likely too high as an intake, in that it would be expected to disrupt sleep when administered with the intention to do so. Specifically, ingestion of caffeine, even at doses below the comparator, can lead to delayed sleep onset and decreases in sleep quality and efficiency, but this is particularly the case when caffeine is consumed near bedtime. Overall, caffeine at doses both above and below the comparator might provide short-term benefits to improve perceived fatigue, but, depending on the dose and timing, may also disrupt sleep, leading to increased fatigue the following day. There were no data for pregnant women. 

The available literature for children and adolescents included in this SR was scant, but the higher-quality studies suggest no major adverse effects on the observed endpoints at doses near or less than 2.5 mg/kg. Above this comparator for all mood endpoints (anger, confusion, anxiety, depression) measured in children and adolescents, it was determined that data were insufficient to develop refined conclusions regarding the potential effects of caffeine. However, the two studies identified that fit the criteria for inclusion suggested no effect of caffeine on mood parameters in adolescents. Regarding headache and sleep, like the other endpoints, it was concluded that there are insufficient quantitative data to evaluate with confidence the effect of caffeine dose on sleep in children and adolescent populations. Based on the limited data, and similar to adults, considerations, such as timing and duration of dose, are likely to be important for these populations. Regarding headache, for children and adolescent populations, there was not enough information, high quality or otherwise, to fully evaluate the appropriateness of the comparator. More targeted research is required to identify sensitive subpopulations in these younger groups, to better quantify the levels at which adverse behavioral effects are observed, and to better understand the link between caffeine consumption and adverse effects. 

Regarding risk-taking behavior, there is sparse evidence that caffeine is associated with an increase in risk-taking behavior in adults. This latter effect is a research area that has seemingly attracted more attention since the work by Nawrot et al. (2003) was published, particularly for younger consumers. Unfortunately, the majority of these studies did not provide quantitative caffeine values for comparison to the comparator value of 400 mg/day. 

Weight of Evidence for Outcome. When the weight of evidence was considered, the comparator, 400 mg caffeine/day, was found to be an acceptable intake that is not associated with significant concern for adverse behavioral effects in adults. However, intake below the comparator may affect some sensitive individuals who are prone to anxiety or sleep disruption. Often, observed effects below the comparator (e.g., anxiety) were limited to subgroups or the timing of dose (e.g., sleep), whereas others were complicated by consumer status (e.g., headache and fatigue). For some endpoints (depression, headache, sleep (subjective), and anger/confusion), there was largely a lack of effects reported, and in some cases, data suggested that intakes higher than the comparator were without effect. There is a moderate to high level of confidence in the body of evidence supporting this conclusion. Confidence was increased by the overall low risk of bias and low level of indirectness, although the variability that was introduced by sensitive subpopulations was a key limitation that precluded a higher level of confidence. It was determined that the evidence base was insufficient to render a conclusion regarding appropriateness of the comparator (2.5 mg caffeine/day) for the potential impacts of caffeine consumption on behavior outcomes in these populations. Overall, the body of literature reviewed for children and adolescents was generally of lower quality when compared to the data for adults. 

#### 3.2.4. Reproductive and Development

**Figure 6:**
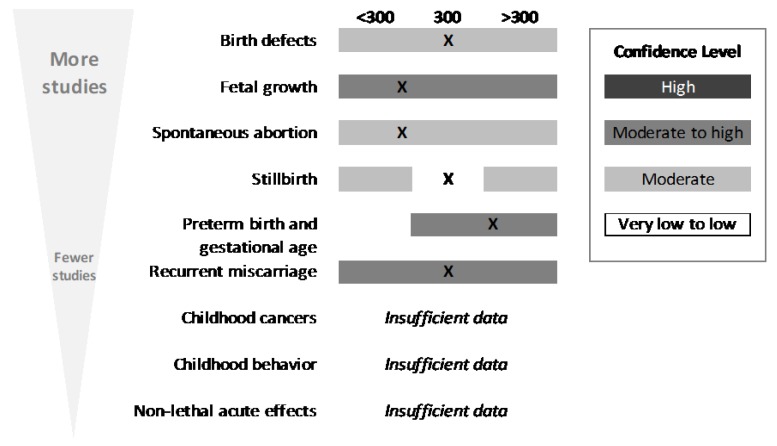
Summary of the spectrum of data and our endpoint conclusions specific to pregnant women, weighted for level of confidence in the body of evidence considering risk of bias, magnitude, consistency, etc. Shading indicates that data reported effects at the corresponding intake level (<300, 300, or >300 mg caffeine/day), and darker shading indicates increased confidence in the body of evidence (from very low to high). X indicates the weight-of-evidence conclusion. Although some effects were seen at intakes lower than 400 mg (e.g., fetal growth, spontaneous abortion), these results did not affect the overall conclusion of the SR due to considerable variability in findings and potential confounding.

Caffeine as a reproductive and/or developmental potential hazard has been and continues to be a point of much discussion. General searching of the internet suggests that pregnant women want to know whether they can have caffeine or not. For this outcome, 58 studies were carried forward as meeting the inclusion criteria of the SR, because they permitted comparison to the Nawrot et al. (2003) [11] conclusions. All of these were focused on adults, with the majority studying pregnant women. As opposed to other outcome areas, a large majority of these were observational, relying on self-reports of caffeine consumption from coffee, soda, and tea in most cases; chocolate, caffeine-containing medications, and energy drinks were the source in a few of the reports. Many of these observational studies, such as the Danish Cohort and Birth Defect Registry, used data from very large, population-based cohorts, meaning that more than 50,000 pregnancies were examined per report. Figure 6 summarizes the key findings for this outcome. 

Common variables accounted for in such analyses included maternal characteristics, such as race, age, weight, BMI, smoking (some using cotinine as a marker), and alcohol consumption. Other factors that were more specific to endpoints of concern were also considered, such as history of pregnancy or miscarriage, partner characteristics, family history of condition, gestational age at birth, and maternal nutrient and supplement intake. Some studies included changes in caffeine consumption during pregnancy as a variable, although most studies did not. Nausea was evaluated as a confounder in most studies, although the extent to which information was collected and incorporated varied. Although confounding factors need to be considered in all epidemiological studies and they were factored into the risk of bias for all endpoints, one unique factor affects reproductive studies in particular. This is a phenomenon known as the “pregnancy signal”: nausea, aversion to smells or tastes, and vomiting are associated with a healthy pregnancy, which then leads to the avoidance of strong smells, including coffee. When not properly controlled for, such avoidance can lead to a misperception that the caffeine (e.g., coffee) is the cause of a pregnancy loss, when in fact, the pregnancy was already in jeopardy, as manifested by the lack of pregnancy signal (i.e., the mother felt no aversion to strong smells) and is correlated with low hormone levels [26,27]. Without specific analysis of coffee aversion, it is difficult to ascertain whether an increased incidence of spontaneous abortion in a study is due to higher caffeine consumption, or if reduced caffeine consumption is occurring in healthier pregnancies due to the pregnancy signal (i.e., reverse causation). 

Many potential adverse-event outcomes were reviewed, and the confidence in the evaluation of the comparator varied. The comparator of 400 mg/day was considered acceptable, with a moderate to high level of confidence on the endpoints of fecundability (the ability to conceive during a given menstrual cycle), fertility, and male reproductive measures. However, due to significant limitations to fully accommodate for the pregnancy signal, the confidence was decreased to a moderate level for the comparator of ≤300 mg/d as an acceptable intake that would be associated with no significant concern for spontaneous abortion, recurrent miscarriage, and stillbirth. Preterm birth and gestational age were considered together, and because the data consistently showed a lack of effects, both above and below the comparator of 300 mg/day, the data suggest that the comparator could be higher. Fetal growth was an endpoint for which the body of evidence was difficult to assess despite there being a large number of studies. The biological significance of the birth-weight changes is evaluated more robustly in studies that assess small for gestational age (SGA) or intrauterine growth restriction (IUGR). These types of studies, as a whole, did not support effects occurring below the comparator of 300 mg/d. However, the low magnitude of effect (measures of association between 1.0 and 2.0 for studies below the comparator)—as well as the observation that, in many cases, the effects were limited to single measures and/or subgroups or were not clinically relevant changes—reduced overall confidence in the data, suggesting that the comparator may be too high. Many types of birth defects have been studied for associations with caffeine exposure: cardiovascular malformations, choanal atresia, cleft lip (with or without cleft palate), cleft palate only, persistent cryptorchidism, and various other individual birth defects, including anotia/microtia, esophageal atresia, diaphragmatic hernia, omphalocele, or gastroschisis. For all of these birth defects, there was no association with maternal caffeine consumption at or above the comparator of 300 mg/day. Additionally, some weak to moderate but inconsistent associations were reported for anorectal atresia, limb defects, and neural tube defects. Thus, although the evidence base is broad with respect to the type of birth defects and underlying etiologies, data were relatively consistent in demonstrating a lack of effects following consumption of caffeine at intakes up to 300 mg/day in healthy pregnant women. Based on the underlying study types (observational), low risk of bias, and consistency in findings, there was a moderate level of confidence in this conclusion. 

Mixed findings for childhood cancers (CNS tumor and childhood leukemia) and their association with maternal consumption of caffeine were attributed to problems with design related to improper control for recall bias (i.e., the phenomenon of individuals experiencing adverse outcomes tending to report more exposure than other individuals, even when no difference may exist). That is, it is generally recognized by epidemiologists that, when asking mothers to recall what they may have ingested during pregnancy after giving birth to a child with a birth defect or disease, they will try to find a cause. For this reason, an alternative study design is for both the case and control populations to have adverse conditions manifested; otherwise, there is a high likelihood of recall bias [28]. Another stronger study design option would be a nested case-control design with prospective assessment of exposure. This topic of confounding was acknowledged by both the authors and observers at the International Agency for Cancer Research (IARC) in the recent review of the potential carcinogenesis of coffee, in which IARC concluded that, overall, coffee drinking was unclassifiable as to its carcinogenicity to humans [12]. The limited number of studies, combined with the significant impact of potential recall bias, precluded the development of a conclusion for this SR but highlights the need for additional research that accommodates this significant bias in the future. 

Another area of much interest in public forums has been prenatal exposure to caffeine and subsequent changes in childhood behavior. Only a few studies were included that related to this endpoint. Because data were limited, and all pertained to different behavioral changes, no conclusion was developed; however, the lack of effects observed in all studies suggests that this is not an endpoint of concern. A number of studies that were included in the review (meeting criteria) fell into the category designated as “other reproductive endpoints”, because only one study was identified per endpoint. These included pregnancy-induced hypertension and/or preeclampsia, and median age at menopause, as well as maternal stress. 

Weight of Evidence for Outcome. The current body of evidence characterizing this endpoint is generally consistent with what was reported by Nawrot et al. (2003); the majority of studies included in the SR do not report reproductive or developmental effects at levels below the relevant comparator. Although effects below 300 mg/day (or 400 mg/day, in the case of males and nonpregnant females) cannot be ruled out with the currently available data, the effects seen at these levels were primarily limited to isolated reports of congenital malformations [29,30] or childhood cancers [31,32], and the findings were of relatively low magnitude. 

#### 3.2.5. Acute Toxicity

Acute effects that are associated with caffeine consumption can include a wide spectrum of symptoms, with headache, nausea, vomiting, fever, tremors, hyperventilation, dizziness, anxiety, tinnitus, and agitation at the milder end of the spectrum [33]. More severe effects resulting from caffeine intoxication can include abdominal pain, altered consciousness, rigidity, and seizures, as well as abnormal heart rhythms and reduced blood flow to the heart [34]. Many of these changes would be expected at very high doses, considering caffeine’s ability to stimulate the central nervous system, among other physiological effects [35]. 

In the SR, we investigated studies addressing death or non-lethal effects following an acute exposure [1]. Acute toxicity as an outcome of interest for the systematic review was defined as abuse, overdose, and potential death due to caffeine. Forty-six full-text papers were reviewed, and 26 were found to meet the criteria, because they permitted comparison to the conclusions of Nawrot et al. (2003) [11]. All 26 were case reports or case series, most of which were associated with emergency department (ED) visits and/or suicide-related events. This was the only endpoint in the systematic review for which case reports were allowed; while the SR authors recognize that these types of reports are not generalizable (because they investigate one incident and not trends within a population), more robust types of data were not identified for this endpoint. 

Of the 26 included, the majority of reports were in adults, with four covering adolescents and two evaluating pregnant women. All of the reports involved very high doses of caffeine (up to 50 g) being delivered over a very short time frame, and in most reports, the authors delivered only brief discussions of the amount of caffeine ingested. In about one-half of the reports, caffeine was consumed as a powder or tablet (sleep aid), and the remaining reports involved energy drinks, with a few involving cola. Coffee and green tea received mentions, but they were not the major sources of caffeine in these intoxications. However, confidence in exposure characterization was low, due to mainly self-reporting with corroboration of friends/relatives as the source. Because Nawrot et al. presented 10 g as the acute lethal dose, 10 g/person was the comparator [11]. 

Key Findings Described in the Body-of-Evidence Characterization: Overall, the current body of evidence related to acute toxicity of caffeine is generally consistent with what was reported by Nawrot et al. (2003) [11], which suggests the potential for death following acute exposures of approximately 10 g of caffeine. The review of the data also supports a lack of nonlethal acute effects at or below exposures of 400 mg/day. However, there is very low to low confidence associated with this conclusion because of the reliance on case reports, ambiguity of exposure levels, and high risk of bias (e.g., case reports are not published when there is no effect). It is notable that each case appeared to have a unique spectrum of adverse events, although vasospasm, seizure, mania, hypokalemia, and muscle weakness were commonly reported. Nearly all of the case reports describing fatalities involved caffeine powder and tablets, whereas the case reports that were associated with other acute (non-lethal) effects generally involved rapid consumption of caffeinated beverages over a short time. 

#### 3.2.6. Caffeine Pharmacokinetics (PK) and Pharmacodynamics (PD)

Simply put, PK refers to the rates of absorption, metabolism, and excretion of caffeine, and PD refers to the effects of caffeine upon the body. In general, the PK/PD of caffeine is well understood; however, we were particularly interested in any new science with respect to differences and similarities between populations of interest, in the context of the five main outcome areas. The review found that most recent research has been in the area of caffeine metabolism focused on how one’s own genetic makeup leads to interindividual differences in how caffeine is handled by the body. 

The most common PK/PD topic reviewed was in relation to how small nucleotide polymorphisms (SNPs) have been characterized, further helping to elucidate individual differences in caffeine metabolism and even consumption practices. This type of work evaluates changes at the allele level in genes and the resultant changes in how one’s body handles exposure to caffeine. As an example, caffeine is a known antagonist of the adenosine receptor, and research has shown that the ADORA2A gene encodes specifically the adenosine A_2A_ receptor; polymorphisms in this gene can affect individual sensitivity to caffeine. Effects can include different sensitivities in feelings of anxiousness following decreased caffeine intake. A fair amount of pharmacogenomic research pertains to two other alleles that are commonly studied: CYP1A2*1F (variant rs762551, genotype AA) and the CYP1A2*1K alleles. These alleles are of interest, because they are associated with increased and decreased caffeine metabolism, respectively. Our findings suggest that epigenetic trends or effects, including further characterizations of SNPs believed to be associated with consumption practices (e.g., self-regulation), as well as specific effects, including several behavioral endpoints (i.e., mood, tolerance, withdrawal), can be important when interpreting overall findings, as well as future endeavors, to characterize sensitive effects or sensitive populations. 

## 4. Discussion

The article, “Systematic Review of the Potential Adverse Effects of Caffeine Consumption in Healthy Adults, Pregnant Women, Adolescents and Children [1]”, summarized herein, provides a comprehensive assessment of evidence in the peer-review literature regarding caffeine safety. Results demonstrated that the conclusions from Health Canada established in 2003 [11] still hold true today. That is, moderate caffeine consumption—up to 400 mg/day in healthy adults, 300 mg/day in healthy pregnant women, or 2.5 mg/kg body weight/day in children and adolescents—is unlikely to be associated with adverse effects. The Special Issue of Nutrients afforded us the opportunity to provide a plain-language summary of the systematic review, thus improving the usability of the SR for health-care professionals and consumers of caffeine. 

Serious considerations were given to the strengths and weaknesses of the systematic review. Key strengths included: (1) Use of the systematic review format based on IOM standards (IOM, 2011) [14]; this format imparts transparency and rigor to the review process (and subsequent confidence in the overall assessment); (2) Assessment of five health outcomes (reproductive and developmental toxicity, behavior, cardiovascular, bone and calcium homeostasis, and acute toxicity); (3) Assessment of four populations (healthy adults, healthy pregnant women, healthy adolescents, healthy children); (4) A large evidence base (>5000 studies considered for eligibility, >381 included across the five outcomes); (5) A multidisciplinary team consisting of subject-matter experts and systematic-review experts; (6) Full transparency in analysis and reporting via the registration of systematic review protocols on PROSPERO, use of the AHRQ Systematic Review Data Repository, and open access to both this summary and the systematic review publication in *Food and Chemical Toxicology*. Additionally, the review sponsor supports a website containing all relevant resources (http://ilsina.org/caffeine-systematic-review-2017). 

Weaknesses of the systematic review included: (1) The large volume of information reviewed precluded the ability to discuss or present all aspects of each study (e.g., all findings, critical appraisal of individual study strengths and limitations); (2) The evidence base was complex and heterogeneous. Study design and reporting varied widely, both within an outcome or endpoint and between outcomes and endpoints; for example, different methods were used to assess caffeine intake, or different approaches were used to measure effects on sleep; (3) Limitations in the overall evidence base did not allow for an assessment of chronic exposures for all endpoints evaluated in the review; for example, data from studies that reported physiological endpoints (e.g., blood-pressure changes) were most often obtained from short-term (often single-exposure) controlled trials; (4) Not all study designs properly controlled for confounding; (5) Various sources of potential bias (pregnancy signal and recall bias) were discussed briefly here, but the reader is also referred to an article in this special issue devoted solely to this topic [27]; (6) Difficulties encountered in characterizing exposure (discussed in more detail below). 

One of the largest areas of uncertainty in the underlying body of evidence assessed herein, and one of much interest to the consumer, is that of exposure. In the case of the SR, confidence in the characterization of exposure for each individual study was not high. Several of the caffeine sources that were included in the SR are complex mixtures with other potentially active compounds, and the amount of caffeine within each source can be highly variable. This is a problem for coffee in particular [4], which was the primary substance evaluated in >20% of studies assessed in this SR. To address this, we attempted to standardize this metric in the SR. It should be noted, however, that the evidence also contains a large number of controlled trials in which exposure was well characterized, although these studies were associated primarily with physiological endpoints. Providing consumers with information that is related to caffeine levels contained in specific products (e.g., better product labeling) will help them to make educated decisions regarding their personal exposure level. 

From recent literature, one can see that other aspects of caffeine consumption are important to consider when determining caffeine safety; for example, the conditions under which various sources of caffeine are consumed and whether caffeine consumption is habitual or not. Our SR evaluated consumption of total caffeine amounts within a day; however, as consistent with the kinetic behavior of caffeine, effects may vary based on how the caffeine is consumed within a day. The most dramatic examples of this are the case studies that report lethality events that are associated with rapid and excessive consumption of capsules or powders (the comparator for lethality (10 g) is equivalent to ~100 cups of coffee). This concern is supported by recent FDA activity designating pure or highly concentrated caffeine in powder or liquid as unlawful (FDA guidance, 2018; https://www.fda.gov/newsevents/newsroom/pressannouncements/ucm604485.htm). Therefore, it is important for the consumer to understand such nuances of exposure. To that end, considering the wide array of caffeine-containing products in the marketplace, and hence, the potential for exposure to caffeine, the consumer’s own perception of the effects of caffeine and self-limitation will remain an important area of research. A recent review by Nehlig (2018) [36] provides insight into consumer self-limiting based on objective (what caffeine does to the body that may not be recognized by the consumer) and subjective effects (the caffeine effects sought by the consumer) of caffeine. Further research will likely continue in the area of interindividual sensitivity and consumption practices, as related to genetic makeup [37]. 

Based on our findings, we would suggest that any discussion with consumers or patients should consider the magnitude and level of the adversity of effects. That is, the pharmacological effects of caffeine are anticipated to cause certain physiological changes and thus require some characterization of the level of significance to health (because not all physiological changes are adverse). An example is that caffeine intake is expected to result in increased alertness, which is often desirable; however, under some conditions (such as prior to bedtime), this is an adverse effect, leading to difficulty sleeping. Another good example is that, while data suggest that caffeine intake can result in changes to heart rate or blood pressure, it is less clear at what level these effects are clinically significant. 

The findings of the SR support the safety of standard consumption practices in the United States, because both mean and upper-end estimated intakes (mean of 165 mg/day and 90th percentile of 395 mg/day, all ages) are below the comparator value evaluated herein. Findings of this assessment, however, also confirm that there is no “bright-line” safe exposure, because potential effects depend on many conditional factors; further, there is some limited evidence that self-regulation reduces consumption [38]. With regard to child and adolescent populations, limited data were identified; however, based on the available studies reviewed, there is no evidence to suggest a need for a change from the recommendation of 2.5 mg/kg body weight/day. Our review supports that additional research would be valuable in this area, as well as in other areas that were identified as having insufficient information—a finding similar to that of other investigators (e.g., Ruxton 2014 [39]). This includes more research on effects in sensitive populations and establishing better quantitative characterization of interindividual variability, as well as subpopulations (e.g., unhealthy populations, those with preexisting conditions), conditions (e.g., co-exposures), and outcomes (e.g., exacerbation of risk-taking behavior) that could render individuals at greater risk relative to healthy adults and pregnant women. 

In addition to the area of self-regulation mentioned above, this work identified other suggested research areas, listed here per outcome area. Bone & calcium: more research in non-adult populations as well as a better understanding of caffeine’s effects on physiology and the role of calcium would be valuable. Cardiovascular disease: a better understanding of dose-response relationships following chronic exposure for some endpoints (e.g., endothelial function and heart rate variability) would be useful. Additionally, for certain physiological effects, research should better characterize what, if any, magnitude of change may be considered harmful. Behavior: more research is necessary on children and adolescents; particularly with regards to caffeine’s effects on sleep and risk-taking behavior. It would also be helpful if more consideration for/or a better understanding of the effects of caffeine withdrawal on these endpoints. The are no data available on pregnant women that fit the quantitative inclusion criteria, so studies designed to account for this would be beneficial. Finally, investigating a better understanding of the effects of caffeine on anxiety and sleep in sensitive subpopulations as well as in individuals with polymorphisms (e.g., ADORA2A) would be of use. Reproductive and developmental: more research is necessary to understand the effect of caffeine on childhood cancer and childhood behavior with properly designed/controlled studies. In addition, more consideration and accounting for the pregnancy signal would be beneficial. Overall, as noted for all outcomes, better exposure characterization in pregnant women to reduce measurement error, which continues to be a major challenge for observational study design, would be valuable. Acute: the main identified research need in this area is improved exposure characterization; testing of blood concentrations would prove valuable.

## 5. Conclusions

In conclusion, the results of the SR support the guidance values that were characterized over a decade ago by Health Canada [11] and reinforce integrative assessments from other authoritative groups (EFSA, 2015) [10]. Recognizing that individuals may differ in their own level of sensitivity to caffeine, our conclusions, as well as those of Health Canada, are intended to provide guidance on safe levels of consumption for healthy consumers. 

## Figures and Tables

**Table 1:** Project team and roles for the systematic review.

Entity	Description	Roles
Scientific Review Team: ToxStrategies	Scientists with a range of expertise (caffeine, toxicology, epidemiology, systematic review, literature searching, etc.)	Develop and perform the systematic review (SR) (consistency in application of SR process, independent assessment, documentation)
Scientific Advisory Board (SAB)	Multidisciplinary experts (systematic review, behavior, cardiovascular, bone & calcium, reproduction & development, acute, pharmacokinetics—PhDs and MDs from academic, private, and clinical practices)	Provide input, review, and approval; develop protocol, conclusions
Sponsor: ILSI North America	Members of the ILSI-North America Working Group (additional funding through two unrestricted grants from the American Beverage Association and the National Coffee Association)	Budgetary
